# A Comprehensive Review of Natural Products against Liver Fibrosis: Flavonoids, Quinones, Lignans, Phenols, and Acids

**DOI:** 10.1155/2020/7171498

**Published:** 2020-10-05

**Authors:** Xiaoqi Pan, Xiao Ma, Yinxiao Jiang, Jianxia Wen, Lian Yang, Dayi Chen, Xiaoyu Cao, Cheng Peng

**Affiliations:** ^1^School of Pharmacy, Chengdu University of Traditional Chinese Medicine, Chengdu 611137, China; ^2^School of Public Health, Chengdu University of Traditional Chinese Medicine, Chengdu 611137, China; ^3^State Key Laboratory of Southwestern Chinese Medicine Resources, Chengdu University of Traditional Chinese Medicine, Chengdu 611137, China

## Abstract

Liver fibrosis resulting from continuous long-term hepatic damage represents a heavy burden worldwide. Liver fibrosis is recognized as a complicated pathogenic mechanism with extracellular matrix (ECM) accumulation and hepatic stellate cell (HSC) activation. A series of drugs demonstrate significant antifibrotic activity in vitro and in vivo. No specific agents with ideally clinical efficacy for liver fibrosis treatment have been developed. In this review, we summarized the antifibrotic effects and molecular mechanisms of 29 kinds of common natural products. The mechanism of these compounds is correlated with anti-inflammatory, antiapoptotic, and antifibrotic activities. Moreover, parenchymal hepatic cell survival, HSC deactivation, and ECM degradation by interfering with multiple targets and signaling pathways are also involved in the antifibrotic effects of these compounds. However, there remain two bottlenecks for clinical breakthroughs. The low bioavailability of natural products should be improved, and the combined application of two or more compounds should be investigated for more prominent pharmacological effects. In summary, exploration on natural products against liver fibrosis is becoming increasingly extensive. Therefore, natural products are potential resources for the development of agents to treat liver fibrosis.

## 1. Introduction

Liver fibrosis is a progressive liver disease that results from a chronic wound healing response to various pathogenic factors, such as chronic infection with hepatitis B virus (HBV)/hepatitis C virus (HCV), alcohol abuse, nonalcoholic fatty liver disease (NAFLD)/nonalcoholic steatohepatitis (NASH), autoimmune hepatitis, hemochromatosis, or primary/secondary biliary cholangitis [1, 2]. Liver fibrosis gradually develops into cirrhosis and causes liver dysfunction with liver failure. This progression is estimated to influence approximately 1% to 2% of the population worldwide. Moreover, over 1 million people will suffer death annually worldwide [3]. It is believed that liver fibrosis is characterized by redundant deposition and intrahepatic accumulation of extracellular matrix (ECM). In the fibrotic liver, quiescent hepatic stellate cells (HSCs) differentiate into proliferative and contractile myofibroblasts. This activation of HSCs plays a primary role in ECM accumulation [4]. The hepatic sinusoid is a well-organized structural and biochemical microenvironment for liver cell survival and communication. This microenvironment is a multidirectional interaction complex with multiple signals or targets [5]. Besides injured hepatocytes, hepatic macrophages, endothelial cells, and lymphocytes are also included due to the abundant blood supply in this microenvironment. Thus, the death of hepatocytes is progressively to release cellular contents and reactive oxygen species (ROS) that activate hepatic immune response containing resident macrophages (Kupffer cells) with excessive proinflammatory factors like TNF-*α*, IL-1*β*, and IL-6 [6]. On the other hand, after sustained liver injury from this progress, liver sinusoidal endothelial cells (LSECs) lose their phenotype and protective properties, promoting angiogenesis and vasoconstriction. It further contributes to repeating inflammation and triggers fibrosis [7]. All these actions will contribute to HSCs activation. HSCs are one of the vital constituents in liver sinusoidal cells and are located between LSECs and hepatocytes. Thus, HSC activation and fibrogenesis are associated with a series of mechanisms, such as transforming growth factor-*β* (TGF-*β*) secretion, autophagy, endoplasmic reticulum stress, oxidative stress, and cholesterol stimulation [4]. Apart from HSC activation, liver fibrosis is widely associated with hepatocyte apoptosis and dysregulation of inflammation. To date, no satisfactory agents for liver fibrosis treatment have been developed due to the complicated progression of liver fibrosis.

In recent years, a number of natural products derived from formulae or composites have drawn much attention as resources for drug discovery [8]. The prevailing natural products involving plants, animals, fungi, and microorganisms are widely used in traditional Chinese medicine (TCM), Ayurveda, Kampo, and Unani. Some natural products have exhibited therapeutic effects on liver diseases for thousands of years. In particular, several kinds of representative natural products, such as flavonoids, quinones, lignans, phenols, and acids, demonstrate potent function in liver protection, cholestasis prevention, NASH inhibition, and hepatocellular carcinoma (HCC) suppression [9, 10]. In addition, natural products that have promising efficacy in preventing fibrosis have been under rapid development [11–13]. Hence, this review provides a comprehensive overview of the potential effects and possible mechanisms of several natural products against liver fibrosis.

## 2. Flavonoids

**Figure 1:**
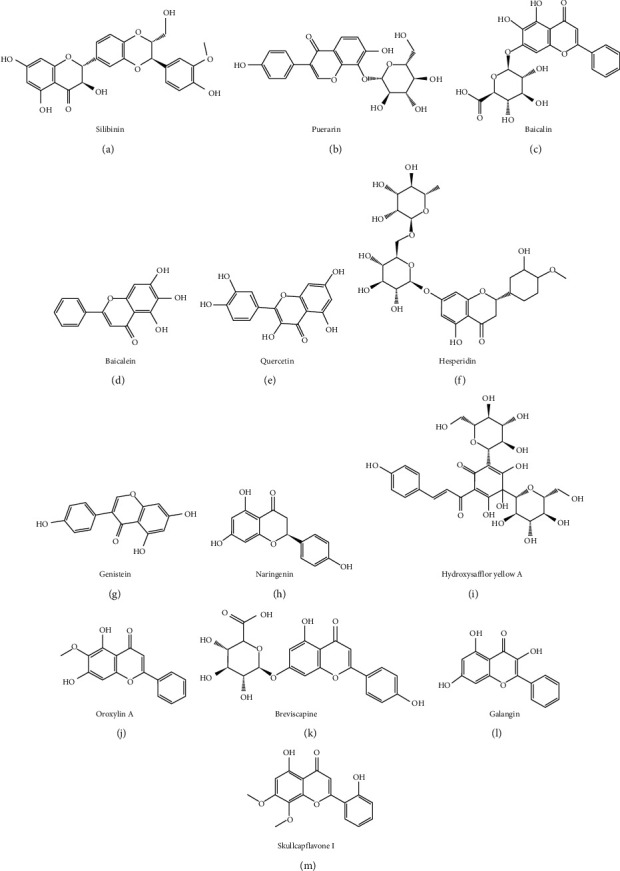
Chemical structures of flavonoid compounds against liver fibrosis.

Flavonoids, a type of compounds with a 2-phenyl flavone structure, are widely distributed in nature, which are characterized by a variety of biological activities, including cardiovascular activity, antibacteria, antivirus, antitumor, antioxidant, anti-inflammatory, and liver protection. Chemical structure of some common flavonoids that have antifibrotic effects is shown in Figure 1.

### 2.1. Silibinin

Silibinin is one kind of flavonolignans isolated from the plant *Silybum marianum* (L.) (milk thistle). It is the major bioactive component and accounts for approximately 60% of silymarin. In recent years, silibinin has been widely applied as a hepatoprotective compound for the treatment of a variety of liver diseases [14]. A team of Italian researchers first confirmed that silibinin at a dose of 100 mg/kg alleviated iron-induced liver fibrosis with mitochondrial dysfunction [15, 16]. Then, another chronic CCl_4_-induced liver fibrosis model was assessed. However, the results indicated that silibinin at 50 mg/kg did not significantly reverse fibrogenic progression [17]. In addition, Wang's team reported that silibinin at 200 mg/kg inhibited thioacetamide- (TAA-) induced liver injury and fibrosis by decreasing *α*-SMA and inflammatory cytokines IL-1*β*, IL-6, and TNF-*α* levels, which was associated with the upregulation of CYP3A via pregnane *X* receptor (PXR) [18]. Silibinin at a dose of 105 mg/kg was effective for preventing hepatic steatosis and fibrosis in a NASH mouse model through regulating lipid metabolism-related gene expression, activating Nrf2, and inhibiting the NF-*κ*B signaling pathway [19]. In an in vitro study, 25–50 *μ*mol/L silybin dose-dependently inhibited the activation of HSC proliferation, cell motility, and ECM deposition through anti-inflammatory activities [20]. Moreover, another recent study indicated that silibinin inhibited LX-2 cell proliferation in a dose- and time-dependent manner and arrested HSC cell cycle by enhancing p53/p27 ratio and inhibiting Akt downstream signaling [21].

### 2.2. Silymarin

Silymarin is a mixture of flavonolignans that mainly contains silybin, silydianin, and silychristin, which are extracted from *Silybum marianum* (L.) [22]. Silymarin is a well-known complex with antioxidant, anti-inflammatory, and immunomodulatory effects on various liver disorders [23]. Silymarin showed antifibrotic effects in a variety of liver fibrosis models, such as alcohol, CCl_4_, TAA, and schistosomiasis-induced models [24–27]. The antihepatic fibrosis mechanism of silymarin seems to be varied. El-Lakkany reported that silymarin at 750 mg/kg alleviated *Schistosoma mansoni*-induced liver fibrosis by downregulating MMP-2 and TGF-*β*1 and upregulating glutathione (GSH) [28]. In a series of CCl_4_-induced liver fibrosis studies, silymarin significantly decreased connective tissue growth factor (CTGF), regulated Kupffer cells, inhibited inflammation via NF-*κ*B signaling, and suppressed the activation of HSCs [29–31]. Moreover, silymarin relieved diet-induced NASH with liver fibrosis by suppressing HSC activation and TNF-*α* release [32]. The combined application of silymarin with other agents, such as caffeine, lisinopril, and taurine, also displayed remarkable antifibrotic effects through the downregulation of LPAR1 and NF-*κ*B expression [33–35].

### 2.3. Puerarin

Puerarin is an isoflavone that is extracted from the plant *Pueraria lobata* (Willd.) Ohwi. Puerarin has abundant pharmacological effects, including cardiovascular protection, neuroprotection, and liver injury prevention [36]. In recent years, puerarin was confirmed to be effective for the treatment of liver fibrosis with multiple target hits. A study suggested that puerarin at doses of 40–80 mg/kg significantly increased the activated HSCs apoptosis and downregulated Bcl-2 expression in a CCl_4_-induced liver fibrosis model [37]. Apart from apoptosis signaling, it is believed that puerarin also alleviates liver fibrosis via inhibition of excessive collagen deposition through peroxisomal proliferator-activated receptor *γ* (PPAR-*γ*) expression and the PI3K/Akt pathway, attenuation of inflammatory response through the TNF-*α*/NF-*κ*B pathway, and suppression of PARP-1 and mitochondrial dysfunction [38–40]. Moreover, puerarin also blocked the TGF-*β*1/Smad signaling pathway in dimethylnitrosamine- (DMN-) induced liver fibrosis and HSC-T6 in a dose-dependent manner [41]. Similarly, puerarin was showed to alleviate TAA-induced liver fibrosis by reducing HSC activation and alleviating ECM expression through the downregulation of the TGF-*β*/ERK1/2 pathway [42]. In addition, the combined application of puerarin and vitamin D showed a significant effect against liver fibrosis through the Wnt/*β*-catenin signaling pathway [43].

### 2.4. Baicalin

Baicalin primarily derived from *Scutellaria baicalensis* Georgi is an active flavonoid compound and is believed to be a specific TCM for treating liver diseases [44]. In the past 15 years, this compound has been widely suggested to have a therapeutic effect on liver fibrosis. Peng's research demonstrated that 70 mg/kg baicalin had a significant effect on CCl_4_-induced liver fibrosis, which was correlated with immunoregulation of the imbalance between profibrotic and anti-fibrotic cytokines, including TGF-*β*1, TNF-*α*, IL-6, and IL-10 expression [45]. Another study also confirmed that baicalin could enhance PPAR-*γ* levels contributing to the downregulation of the TGF-*β*1 signaling pathway and the suppression of HSC activation to play antifibrotic effect [46]. Baicalin also decreased liver fibrosis progression with marked inhibition of *α*-SMA, TGF-*β*1, and Col1A1 expression in a NASH model [47]. Furthermore, baicalin at doses of 50–150 *μ*mol/L inhibited platelet-derived growth factor-B subunit homodimer- (PDGF-BB-) induced HSC-T6 activation via the miR-3595/ACSL4 axis in vitro [48]. Other systematic studies indicated that baicalin combined with rosmarinic acid suppressed fibrotic effects via PPAR-*γ* in HSCs [49].

### 2.5. Baicalein

Baicalein is another active flavonoid from *Scutellaria baicalensis* Georgi. Baicalein possesses hepatoprotective activities via a variety of biological properties [50]. Treatment with 20–80 mg/kg baicalein dose-dependently decreased hydroxyproline and MMPs in CCl_4_-induced liver fibrosis, which was related to reduction of inflammation, liver structural repair, and protein synthesis inhibition of the PDGF-*β* receptor [51]. Moreover, 3–30 *μ*mol/L baicalein presented potent antiproliferative effect in HSCs stimulated with PDGF-BB-, indicating that baicalein could be regarded as a potential antifibrotic drug [52].

### 2.6. Hesperidin

Hesperidin is a flavanone glycoside that is widely found in citrus fruits and has various biological activities, such as neuroprotection, liver protection, anticancer effects, and inflammatory regulation [53, 54]. Hesperidin was found to be effective for liver fibrosis treatment based on a series of liver fibrosis models. Hesperidin at doses of 100 and 200 mg/kg demonstrated antifibrotic effects by reducing HSC activation in DMN-induced fibrosis [55]. In a CCl_4_-induced liver fibrosis model, hesperidin at 200 mg/kg decreased lipid peroxidation, NF-*κ*B, TGF-*β*, CTGF, and IL-1*β* and increased IL-10 expression [56]. Moreover, hesperidin prevented from the progression of BDL-induced liver fibrosis through inhibition of TGF-*β*1/Smad signaling pathway-mediated ECM deposition and apoptosis [57]. A similar study in vitro indicated that hesperidin inhibited HSC-T6 proliferation and activation by modulating the TGF-*β*1/Smad signaling pathway [58].

### 2.7. Quercetin

Quercetin, a well-known flavonoid, is widely found in various vegetables and fruits, including onion, mulberry, tomato, apples, and tea [59]. Quercetin is thought to effectively suppress liver fibrosis involving multiple mechanisms. A study from Lee first reported that administration of quercetin at 10 mg/kg markedly prevented from DMN-induced liver fibrosis by reducing TGF-*β*1 expression [60]. Quercetin at doses of 5–200 mg/kg significantly alleviated liver fibrosis induced by CCl_4_ through regulating inflammation involving NF-*к*B and MAPK signals and protecting from apoptosis via inhibiting proapoptotic gene Bax and improving antiapoptotic gene Bcl-2 expression [61]. Quercetin also reduced autophagy via the TGF-*β*1/Smad and PI3K/Akt pathways, improving HSC apoptosis and activating MMPs, as well as inhibiting macrophage infiltration and modulating M1 macrophage polarization by regulating the Notch1 signaling pathway [62–64]. In addition, quercetin modulated the HMGB1-TLR2/4-NF-*κ*B signaling pathways to inhibit HSC activation in CCl_4_-induced liver fibrosis [65]. In BDL-induced liver fibrosis rats, quercetin markedly inhibited ROS-associated inflammation and ameliorated insulin resistance via the STAT3/SOCS3/IRS1 signaling pathway [66]. In vitro research also showed that liver steatosis, inflammatory cell accumulation, oxidative stress, and liver fibrosis were totally or partially prevented by treatment with quercetin [67].

### 2.8. Genistein

Genistein is an isoflavone that is found in several soy products and has potential therapeutic effects on a multitude of diseases [68]. Increasing evidence has shown that genistein is specifically hepatoprotective [69–71]. Ganai reported that genistein at a dose of 5 mg/kg ameliorated D-GalN-induced liver fibrosis by inhibiting the TGF-*β*/Smad2/3 signaling pathway [72]. Genistein also significantly attenuated CCl_4_-induced and BDL-induced liver fibrosis [73, 74]. Schistosomiasis-induced liver fibrosis was also markedly reversed by genistein through the NF-*κ*B signaling pathway [75, 76]. Furthermore, genistein significantly decreased hepatic injury and fibrosis induced by chronic alcohol after treatment with 0.5–2 mg/kg [77]. Genistein inhibited proliferation and activation of HSCs as a tyrosine protein kinase inhibitor in vitro [78]. In addition, several studies conducted by Liao indicated that genistein combined with taurine and epigallocatechin gallate had a significantly preventive effect on liver fibrosis via protein metabolism, as well as glycolysis, gluconeogenesis, and ribosomal regulation [79–84].

### 2.9. Naringenin

Naringenin is a specific flavanone that is distributed in several plants, such as cherries, cocoa, grapes, tangelos, and lemons [85]. Naringenin displays wide preventative properties in liver damage induced by alcohol, CCl_4_, lipopolysaccharide, and heavy metals [86]. For instance, naringenin at doses of 20 and 50 mg/kg prevented from liver fibrosis induced by DMN [87]. Naringenin at a dose of 100 mg/kg significantly reversed CCl_4_-induced liver fibrosis by blocking the TGF-*β*-Smad3 and JNK-Smad3 signaling pathways [88]. In addition, in vitro research revealed that naringenin exerted antifibrotic effects by downregulating Smad3 and TGF-*β* phosphorylation in activated HSCs [89].

### 2.10. Hydroxysafflor Yellow A

Hydroxysafflor yellow A, a flavonoid compound extracted from *Carthamus tinctorius* L., has received extensive attention and possesses broad pharmacological activities [90]. Zhang's research revealed that 5 mg/kg hydroxysafflor yellow A attenuated liver fibrosis by downregulating TGF-*β*1, inhibiting phosphorylation of Smad4, and suppressing the ERK5 signaling pathways [91, 92]. The antifibrotic effect of hydroxysafflor yellow A was also correlated with the regulation of the PPAR-*γ*/p38 MAPK signaling pathway and a reduction in oxidative stress [93, 94]. Hydroxysafflor yellow A was further proven effective in stimulating PPAR activity, reducing cell proliferation, and suppressing ECM synthesis in vivo and in vitro [95]. Apart from these signaling pathways, hydroxysafflor yellow A also induced apoptosis by blocking ERK1/2 kinase in primary HSCs [96].

### 2.11. Oroxylin A

Oroxylin A is the main bioactive flavonoid extracted from *Scutellaria baicalensis* Georgi. Many studies have shown that oroxylin A demonstrates a variety of pharmacological properties against oxidative stress, inflammation, metastasis, and hepatosteatosis [97–100]. A series of studies showed that oroxylin A ameliorated hepatic fibrosis by inhibiting HSC activation. Zheng revealed that continuous treatment with oroxylin A at 20 mg/kg reduced CCl_4_-induced liver fibrosis. Oroxylin A significantly inhibited the reversion of lipid droplets by reducing ROS-dependent adipose triglyceride lipase in activated HSCs [101]. Angiogenesis of liver sinusoidal endothelial cells is closely related to liver fibrosis. Oroxylin A prevented from angiogenesis of LSECs in liver fibrosis via inhibition of the YAP/HIF-1*α* signaling pathway [102]. Moreover, inhibition of glycolysis-dependent contraction via suppression of lactate dehydrogenase-A (LDH-A) and induction of apoptosis via endoplasmic reticulum (ER) stress in HSCs were involved in antihepatic fibrosis effects of oroxylin A [103, 104]. Apart from these signaling pathways, oroxylin A also alleviated CCl_4_-induced liver fibrosis by activating autophagy signaling [105].

### 2.12. Anthocyanins

Anthocyanins are compounds that are widely isolated from fruits and dietary supplements, such as grapes, blueberries, purple-fleshed sweet potatoes, and peanuts. Anthocyanins are believed to have many health-promoting benefits with prominent anti-inflammatory, antioxidant, and immunomodulatory effects [106]. Choi reported that treatment with 50–200 mg/kg anthocyanins significantly decreased DMN-induced enhancement of *α*-SMA and collagen types I and III levels in a liver fibrosis model, as well as TNF-*α* and TGF-*β* [107]. Another study further indicated that anthocyanins were efficient in attenuating HSC-T6 proliferation, which was associated with blocking the PDGFR*β* signaling pathway and suppressing Akt and ERK1/2 activation and *α*-SMA expression [108]. Anthocyanins alleviated HSC activation and liver fibrosis in both HSCs-T6 cells and CCl_4_-treated rats. Anthocyanins from blueberries improved liver function and liver fibrosis by regulating histone acetylation in rats with hepatic fibrosis [109]. In addition, a study revealed that anthocyanins ameliorated liver fibrosis in a network manner by manipulating oxidative stress, inflammation, and HSC activation to remodel ECM deposition [110]. Cyanidin-3-O-*β*-glucoside is a classical anthocyanin with potent antifibrotic activity. A series of studies from Ling's team suggested that 800 mg/kg dietary cyanidin-3-O-*β*-glucoside alleviated liver fibrosis by suppressing inflammatory factors, such as TNF-*α*, IL-6, and IL-10. The progression of HSC activation was also blocked by cyanidin-3-O-*β*-glucoside through significant inhibition of HSC proliferation and migration [111, 112].

### 2.13. Other Flavonoids

There are other flavonoid compounds that improve liver fibrosis, such as breviscapine, galangin, and skullcapflavone I. A study from Liu indicated that 15–30 mg/kg breviscapine dose-dependently reduced collagen deposition and narrowed fibrotic area induced by CCl_4_, which was partially related to inhibition of inflammatory apoptotic response and ROS generation [113]. Treatment with 20–80 mg/kg galangin also significantly reversed CCl_4_-induced rat liver fibrosis by inhibiting HSC activation and proliferation [114]. In early research, skullcapflavone I could induce apoptosis of HSCs to exert antifibrotic effects by activating caspase-3 and caspase-9 [115].

## 3. Quinones

**Figure 2:**
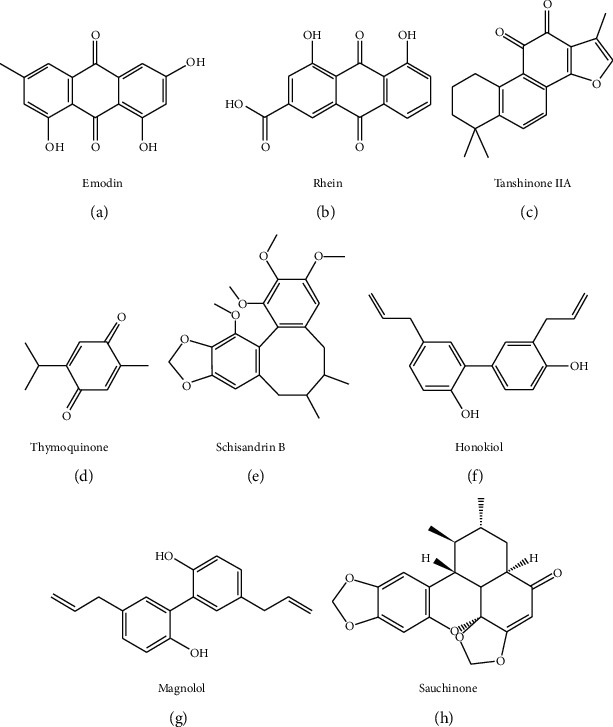
Chemical structures of quinone and lignan compounds against liver fibrosis.

Quinones belong to aromatic organic compounds with cyclic diketone structure of six-carbon atom containing two double bonds, including emodin and rhein, tanshinone IIA, and thymoquinone (Figures 2(a)–2(d)). Anthraquinone and its derivatives are particularly important in TCM. These quinones have various physiological activities, such as excretion promotion, diuretic effect, antibacteria and antivirus, hemostasis, and spasmolysis.

### 3.1. Emodin and Rhein

Emodin and rhein are the main bioactive anthraquinones derived from the rhizome of *Rheum palmatum* L. . Treatment with 20–80 mg/kg emodin significantly alleviated hepatic fibrosis [116]. Zhao's research indicated that the mechanism of emodin against liver fibrosis was partially related to a reduction in the infiltration of Gr1hi monocytes [117]. Moreover, emodin also protected against liver fibrosis and HSC activation by reducing TGF-*β*1 and Smad4 expression and inhibiting tissue inhibitor of metalloproteinases-1 (TIMP-1) expression, and epithelial-mesenchymal transition [118–120]. In addition, 25 and 100 mg/kg rhein inhibited liver fibrosis induced by CCl_4_/ethanol in rats, which possibly benefited from antioxidant and anti-inflammatory activities of rhein. The antifibrotic effect of rhein was also related to inhibition of TGF-*β*1 [121].

### 3.2. Tanshinone IIA

Tanshinone IIA is the main active diterpene quinone phytochemical extracted from *Salvia miltiorrhiza* Bunge (Labiatae). Tanshinone IIA has been commonly used for the treatment of liver diseases, such as hepatic injury, NASH, hepatic fibrosis, and HCC in recent years [122]. A systematic review summarized that tanshinone IIA at doses of 2–200 mg/kg significantly improved liver function in CCl_4_, DMN, TAA, and pig serum-induced liver fibrosis. Various mechanisms, such as reduction of inflammation, inhibition of immunity and antiapoptotic processes, and induction of apoptosis to inhibit HSC activation, are involved in antifibrosis effects of tanshinone IIA [123]. For example, tanshinone IIA enhanced apoptosis of HSCs by promoting the ERK-Bax-caspase signaling pathway via the C-Raf/prohibition complex [124]. Tanshinone IIA also inhibited cell proliferation by arresting the cell cycle in S phase in activated rat HSCs [125]. Moreover, the antifibrotic effect of tanshinone IIA was proved by the team's further research. Su and Zhang found that tanshinone IIA was able to significantly alleviate ECM accumulation HSC proliferation as well as activation. The molecular mechanism involved MAPK, Wnt, and PI3K/Akt signaling pathways via inhibiting c-Jun, p-c-Jun, c-Myc, CCND1, MMP9, P65, p-P65, PI3K, and P38 [126]. In addition, tanshinone IIA could also work effectively in combination application such as Fuzheng Huayu formula, a well-known Chinese patent medicine used for liver fibrosis, according to a series of high-quality studies [127–129].

### 3.3. Thymoquinone

Thymoquinone is the active ingredient from *Nigella sativa* plants. This compound has a protective effect against various types of liver diseases primarily due to its anti-inflammatory and antioxidant properties [130]. A study from Bai demonstrated that 20 and 40 mg/kg thymoquinone significantly enhanced the phosphorylation of AMPK and LKB-1. Thus, regulation of the AMPK signaling pathway might be involved in the inhibitory effect of thymoquinone on ECM accumulation and TAA-induced liver fibrosis [131]. Another study indicated that thymoquinone at 25 mg/kg alleviated CCl_4_-induced liver fibrosis through markedly suppressing activated rat HSCs and LX2 cells by inhibiting the proinflammatory response [132]. Furthermore, the antifibrosis mechanism of thymoquinone was correlated with blocking TLR4 expression and inhibiting the phosphorylation of PI3K in activated HSCs [133]. The combination of thymoquinone and vitamin D exhibited marked antifibrotic effects by downregulating TGF-*β*1, IL-6, and IL-22 and upregulating MMP-9 and IL-10 [134].

## 4. Lignans

Lignans are a kind of natural compounds synthesized by the polymerization of two phenylpropanol derivatives (C6–C3 monomer), such as schisandrin B, honokiol and magnolol, and sauchinone (Figures 2(e)–2(h)). Most of them are free, while a few glycosides are combined with sugar and exist in the wood and resin of plants. Lignans have the functions of antitumor, liver protection, antioxidation, antivirus, and neuromodulation.

### 4.1. Schisandrin B

Schisandrin B is an important bioactive lignan derived from a well-known herbal medicine named *Schisandra chinensis* that has been used for liver protection in recent years. Schisandrin B at 25 and 50 mg/kg significantly attenuated liver damage and liver fibrosis progression in CCl_4_-treated rats. Schisandrin B also markedly suppressed HSC-T6 activation. Schisandrin B could exert antifibrotic effects by increasing the Nrf2-ARE signaling pathway and decreasing the TGF-*β*/Smad signaling pathway [135]. Schisandrin B at 2.5 and 5 *μ*mol/L also attenuated lipopolysaccharide- (LPS-) induced HSCs activation by upregulating Nrf-2 expression [136]. Moreover, transcriptomic analyses were also applied for mechanistic investigation. The results indicated that metabolic pathways, CYP450 enzymes, and the PPAR signaling pathway were the major target pathways of schisandrin B [137].

### 4.2. Honokiol and Magnolol

Honokiol and magnolol are the main bioactive lignans isolated from *Magnolia officinalis* Rehd. et Wils [138]. In recent years, a number of studies have focused extensive attention on the hepatoprotective effect of honokiol and magnolol. Treatment with honokiol at 10 mg/kg alleviated ConA-induced liver fibrosis by downregulating hydroxyproline, *α*-SMA, and collagen fiber deposition, which was associated with restoring antioxidant defense, regulating inflammatory cascades, and inhibiting the TGF-*β*/Smad/MAPK signaling pathway [139]. In vitro research showed that 12.5–50 *μ*mol/L honokiol induced apoptotic death in activated rat HSCs through the release of mitochondrial cytochrome C [140]. Magnolol also attenuated ConA-induced liver fibrosis and suppressed human LX2 HSC activation, which was closely related to inhibiting Th17 cell differentiation by suppressing IL-17A generation [141]. In addition, other honokiol derivatives, such as 4′-O-methylhonokiol, also prevented from HSC activation and induced apoptosis via regulation of Bak1 and Bcl-2 expression [142].

### 4.3. Sauchinone

Sauchinone is a bioactive lignan that is mainly extracted from *Saururus chinensis* and has been widely used for treating fever, edema, jaundice, and several inflammatory diseases [143]. A team from Korea investigated the effect of sauchinone on liver fibrosis. The results showed that sauchinone at 10 and 20 mg/kg alleviated CCl_4_-induced liver fibrosis and inhibited TGF-*β*1-induced HSC activation, which might be associated with suppressing autophagy and oxidative stress in HSCs [144]. Sauchinone also has liver protection effect to resist liver fibrosis. For instance, sauchinone protected the liver from toxicity induced by iron accumulation by regulating LKB1-dependent AMPK activation [145].

## 5. Phenols and Acids

**Figure 3:**
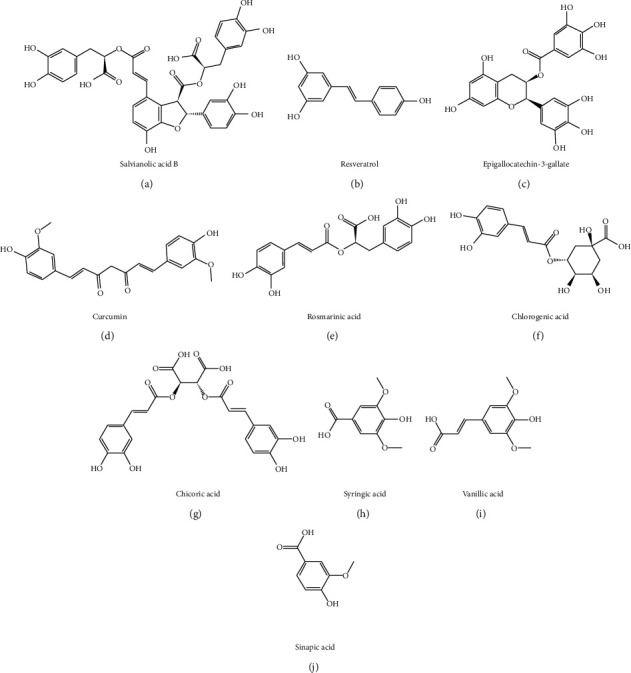
Chemical structures of phenol and acid compounds against liver fibrosis.

One or more hydroxyl groups are directly connected to the benzene ring in the chemical structure of phenolic compounds with weak acid, which is easy to be oxidized in the environment. Chemical structure of several representative phenols and acids is shown in Figure 3. Phenols always have the functions of antioxidation, anti-inflammatory, antiviral, and antitumor.

### 5.1. Salvianolic Acid B

Salvianolic acid B (SA-B) is a water-soluble phenolic acid isolated from *Salvia miltiorrhiza* Bunge. SA-B is a promising compound for the treatment of liver fibrosis according to reports in vivo and in vitro. Previous studies showed that SA-B significantly inhibited HSC activation by modulating the MAPK and Smad3 signaling pathways and ROS accumulation to reduce matrix collagen deposition and TGF-*β*1 expression [146–148]. Inhibition of ERK, MEF2, and p38 MAPK-MKK3/6 signaling was also involved in the antifibrotic effect of SA-B both in vivo and in vitro [149, 150]. SA-B prevented from liver fibrosis in CCl_4_- and DMN-treated models, which were probably related to downregulating the Ang II signaling pathway by AT1R, ERK, and c-Jun phosphorylation and by regulating the NF-*κ*B/IkB*α* signaling pathway [151, 152]. Yu reported that SA-B also inhibited the microRNA-17-5p-activated Wnt/*β*-catenin pathway and modulated lincRNA-p21 expression in HSC activation [153, 154]. In addition, a series of studies testified that the regulation of TGF-*β*/Smad, MAPK, TbR-I kinase, and microRNA-152 targets contributed to the antifibrotic effect of SA-B [155–157].

### 5.2. Resveratrol

Resveratrol is a natural polyphenol that is widely found in grapes, peanuts, berries, and nuts. A variety of studies have focused on the antifibrotic effect of resveratrol in recent years. Treatment with 10–50 mg/kg resveratrol prevented from liver fibrosis induced by CCl_4_. The reduction in NF-*κ*B activation and Akt/NF-*κ*B signaling pathways might contribute to the antagonistic effect of resveratrol on liver fibrosis [158–160]. Another study indicated that resveratrol alleviated fibrosis by producing IL-10 through polarization of macrophages in a CCl_4_-induced model [161]. Resveratrol also significantly inhibited DMN-induced liver fibrosis at doses of 10–20 mg/kg through improving antioxidant defense and alleviating oxidative stress [162, 163]. In addition, resveratrol was proven to be effective in *N*′-nitrosodimethylamine- (NDMA-) induced fibrosis via reducing oxidative damage and resisting HSC activation [164]. Resveratrol demonstrated an antifibrotic effect by modulating alpha fetoprotein transcriptional levels and normalization of protein kinase C (PKC) responses in a TAA-treated model [165]. Resveratrol also had a potent effect on fibrosis induced by NASH and *Schistosoma japonicum* infection [166, 167]. Similar studies in vitro verified that resveratrol inhibited the activation of HSCs by modulating PPAR-*γ*/SIRT1 signals and blocking NF-*κ*B activation, as well as PI3K/Akt phosphorylation [168, 169]. Resveratrol also induced HSC apoptosis by modulating autophagy/mitophagy and mitochondrial biogenesis [170].

### 5.3. Epigallocatechin-3-Gallate

Epigallocatechin-3-gallate (EGCG) is a major polyphenol that accounts for 10%–15% of the components in green tea [166]. EGCG is also a well-known antioxidant with potent activity in various diseases [171]. Several studies revealed that 25–300 mg/kg EGCG attenuated CCl_4_-induced liver fibrosis partially through inhibiting HSC activation and targeting MMP-2 via the modulation of membrane type 1-MMP activity [172]. The reduction in oxidative stress and proinflammatory response may also contribute to the effect of EGCG against liver fibrosis [173]. EGCG effectively altered fibrogenesis by blocking ERK and Smad1/2 phosphorylation, as well as targeting PDGFR*β* and IGF-1R [174, 175]. Moreover, EGCG markedly inhibited BDL-induced liver fibrosis and TGF-*β*1-stimulated LX-2 cells and downregulated profibrotic markers. The antifibrotic effect of EGCG was related to inhibiting the PI3K/Akt/Smad signaling pathway and modulating mitochondrial oxidative stress and inflammation [176, 177]. Arffa reported that EGCG reduced TAA-induced fibrosis and inhibited osteopontin by upregulating miR-221 [178]. Furthermore, EGCG treatment counteracted the activated effects of the TGF/Smad, PI3K/Akt/FoxO1, and NF-*κ*B signaling pathways to alter hepatic fibrosis in NAFLD models [179]. Studies have revealed that EGCG inhibits HSC activation in primary rat HSCs, TWNT-4 cells, and LI90 cells. A study by Sakata revealed that EGCG inhibited PDGF-BB-induced cell proliferation of LI90 cells by blocking PDGF-BB binding to its receptor in a noncompetitive manner [180]. Moreover, Nakamuta found a similar antifibrotic effect of EGCG in TWNT-4 human HSCs by regulating ERK1/2, c-Jun kinase, and p38 via the suppression of Rho signaling pathways [181, 182]. Further studies also suggested that EGCG suppressed MMP-2 activation and HSC invasiveness and induced *de novo* synthesis of GSH [183, 184]. In addition, the combination therapy of EGCG with taurine, genistein, or atorvastatin also demonstrated a significant antifibrotic effect [78, 185].

### 5.4. Curcumin

Curcumin is a well-known polyphenolic compound that is mainly isolated from *Curcuma longa* [186]. In the past 20 years, accumulating evidence in vivo and in vitro has shown that curcumin is a promising agent for liver fibrosis treatment. Studies indicated that curcumin exerted antifibrotic effect via networks and multiple signals. For example, curcumin suppressed HSCs by targeting PPAR-*γ* signaling in vitro [187, 188]. The increase in PPAR-*γ* might inhibit the gene expression of receptor for advanced glycation end-products (RAGE) and attenuate oxidative stress in HSCs [189]. Moreover, inhibition of srebp-2 by modulating specificity protein-1 and suppressing inflammatory cytokines, including IL-6, TNF-*α*, and INF-*γ*, also contributed to antihepatic fibrosis effect of curcumin [190, 191]. Other studies revealed that curcumin could promote apoptosis of activated HSCs by upregulating caspase-3, Bax, and p53 and downregulating Bcl-2 to alleviate liver fibrosis [192–195]. Wu's team suggested that curcumin primarily attenuated liver fibrosis by modulating immune system by blocking Gr1hi monocytes via MCP-1 and reducing Ly6Chi monocyte infiltration via Kupffer cell activation [196, 197]. In addition, curcumin also reversed aberrant methylation in liver fibrosis in vivo and in vitro [198]. Several studies from Zhou's group indicated that curcumin inhibited HSCs by affecting the *β*-catenin signaling pathway and regulating the Shh-associated delta-like homolog 1 (DLK1) signaling pathway [199, 200]. Furthermore, curcumin could exert antifibrotic activity through regulating the AMPK/PGC-1*α* axis to inhibit HSC activation [200]. Another team found that the core mechanism by which curcumin affected liver fibrosis was inhibiting the TGF-*β*1/Smad signaling pathway and CTGF expression [201]. The inhibition of the TGF-*β*/Smad signaling pathway was also observed in alcohol-induced hepatic fibrosis [202]. In addition to the above signals, curcumin also reduced HMGB1, TLR4, and TLR2 expression in fibrogenesis, while ameliorating intrahepatic angiogenesis and capillarization of the sinusoids during liver fibrosis [203, 204]. A series of studies revealed that curcumin alleviated ECM deposition and regulated HSC senescence by suppressing cannabinoid receptor type-1 (CBR1) signaling, activating the PPAR-*γ*/p53 signaling pathway, inhibiting sinusoidal angiogenesis, and activating Nrf2 to induce the lipocyte phenotype in HSCs [205, 206]. Furthermore, NK cells and Hedgehog signaling are also regulated by curcumin to inhibit fibrotic progression [207, 208]. A study initially verified that curcumin protected against CCl_4_-induced hepatic fibrosis by suppressing HIF-1*α* via the ERK-dependent signaling pathway [209]. Another study further showed that curcumin prevented from the activation of HSCs by blocking the succinate/HIF-1*α* signaling pathway [210]. Several other signals, including the modulation of CB1 receptors, IRS1, SOCS3, and STAT3 targets, MyD88 pathway, CXCL12/CXCR4 biological axis, Plin5 gene expression, and LD formation, were also involved in various mechanisms underlying antihepatic fibrosis effect of curcumin [211–215].

### 5.5. Rosmarinic Acid

Rosmarinic acid is a natural polyphenolic antioxidant derived from a variety of common herbal plants [216]. Studies indicated that rosmarinic acid might have a therapeutic effect on liver fibrosis. For instance, a study from Li revealed that 2.5–10 mg/kg rosmarinic acid significantly suppressed TGF-*β*1 and CTGF expression in CCl_4_-induced liver fibrosis. Rosmarinic acid markedly inhibited HSC proliferation and activation by decreasing *α*-SMA, TGF-*β*1, and CTGF levels in HSC-T6 cell line [217]. Similarly, El-Lakkany reported that rosmarinic acid inhibited liver fibrosis progression by inhibiting HSC activation and proliferation by initiating apoptosis-related signals in a TAA-induced model and HSC-T6 cells [218]. Furthermore, rosmarinic acid stimulated the activity of ARE promoter through enhancing GSH level and removing ROS by stimulating Nrf2 translocation into the nucleus, GSH enhancement, and subsequent GCLC upregulation. Thus, ROS elimination by rosmarinic acid directly inhibited NF-*κ*B-dependent MMP-2 expression and suppressed HSC activation [219]. In addition, rosmarinic acid is also the active ingredient in a potent antifibrotic herbal medicine named Yang-Gan-Wan. Rosmarinic acid and Yang-Gan-Wan are effective against liver fibrosis by suppressing the canonical Wnt signaling pathway and reducing PPAR-*γ* in HSCs [47].

### 5.6. Chlorogenic Acid

Chlorogenic acid is one of most plentiful phenolic acids and is widely distributed in various fruits, plants, and vegetables [220]. Many studies have confirmed that chlorogenic acid is specifically potent in liver protection. A series of studies indicated that 30 and 60 mg/kg chlorogenic acid significantly repressed liver fibrosis induced by CCl_4_ by inhibiting HSC activation and downregulating fibrogenetic factors [221]. Moreover, chlorogenic acid could present the hepatoprotective effect through inhibiting the TLR4/MyD88/NF-*κ*B signaling pathway by downregulating several cytokines, such as IL-1*β*, IL-6, TNF-*α*, iNOS, and COX-2 [222]. In addition, a study reported that NOX subunits expression, ROS generation, and the phosphorylated levels of p38 and ERK1/2 decreased in HSCs, while Nrf2 and Nrf2-regulated antioxidant genes (HO-1, GCLC, and NQO-1) increased in liver tissues after chlorogenic acid intervention. Therefore, chlorogenic acid could prevent from liver fibrosis by activation of Nrf2 pathway and inhibition of NOX/ROS/MAPK pathway [223]. Another research group revealed that chlorogenic acid displayed significant antifibrotic effects on the hepatic stellate LX2 cell line and *Schistosome*-infected mice mainly through IL-13/miR-21/Smad7 signaling interaction [224]. Chlorogenic acid also blocked the miR-21-regulated TGF-*β*1/Smad7 signaling pathway by decreasing the expression of *α*-SMA, TGF-*β*1, and collagen I in CCl_4_-induced liver fibrosis and LX2 cells [225].

### 5.7. Other Phenols and Acids

Apart from the above compounds, there are several other phenols and acids used for liver fibrosis treatment, such as chicoric acid, syringic acid, vanillic acid, and sinapic acid. Chicoric acid is a natural phenolic acid that is mainly isolated from chicory and *Echinacea* plants [226]. A study from Kim suggested that 10–30 mg/kg chicoric acid significantly reduced liver fibrosis through downregulating *α*-SMA, TGF-*β*1, and collagen expression in NASH mice. Inhibition of NF-*κ*B-regulated inflammatory response, suppression of AMPK-mediated lipid/triglyceride accumulation, and enhancement of Nrf2 antioxidant defense system might contribute to the effect of chicoric acid against NASH and fibrosis [227]. Syringic acid and vanillic acid are two phenolic compounds found in fruits and vegetables, which markedly suppressed collagen accumulation and decreased the hepatic hydroxyproline content in CCl_4_-induced liver fibrosis [228, 229]. Moreover, these compounds inhibited the activation of cultured HSCs but did not influence hepatocyte viability [230]. Thus, treatment with syringic acid and vanillic acid could suppress the progression of fibrosis during chronic liver injury. Sinapic acid is an orally bioavailable phenolic acid from spices, citrus and berry fruits, and vegetables with wide pharmacological effects [231]. Shin revealed that 10 and 20 mg/kg sinapic acid significantly ameliorated of *α*-SMA, TGF-*β*1, and col I expression in DMN-induced liver fibrosis, which might be relevant to antioxidant effect and suppression of NF-*κ*B and TGF-*β*1 signals [232].

## 6. Outlook and Conclusions

### 6.1. Limitations and Outlook

**Figure 4:**
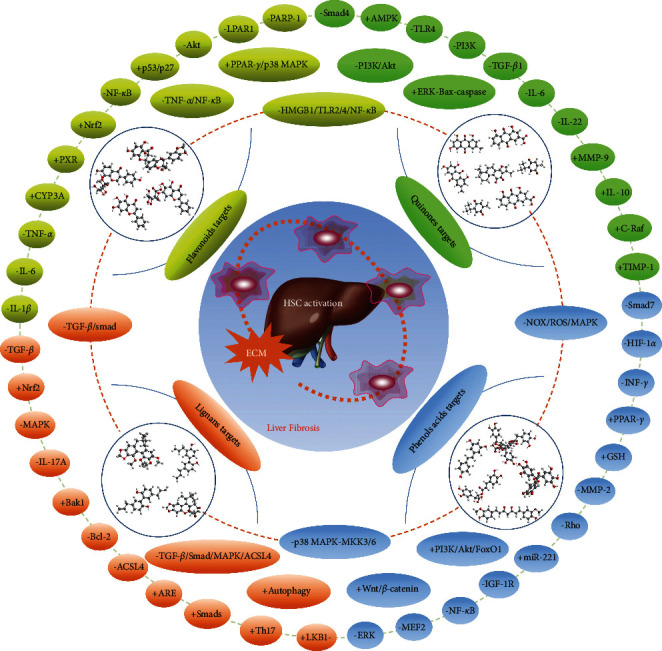
The interaction network mechanism of natural products against liver fibrosis.

Growing evidence suggests that liver fibrosis is a complicated process involving multiple dysfunctions of sinusoidal cells [5]. HSC activation and ECM deposition are thought to be the key markers of fibrogenesis. Recently, many attempts have been made to explore the antifibrotic effects of some natural products to promote drug discovery. Previous study by us reviewed the risk components of TCM-induced liver injury, including alkaloids, glycosides, toxic proteins, terpenoids and lactones, anthraquinones, and heavy metals [233]. In this review, we summarized only four kinds of natural compounds that exert potential antifibrotic function, especially their biological activities and potential mechanisms against liver fibrosis. However, three aspects should be noted. First, the transition from bench to bedside is limited for most natural products. Silymarin and silybin are currently used in the clinic for liver protection during fibrosis in China [234, 235]. However, other promising compounds, such as genistein, quercetin, resveratrol, and curcumin, are still waiting for further clinical confirmation or more evidence of efficacy. Secondly, most of these compounds show antifibrotic effects via multiple targets or signaling pathways (Figure 4). For instance, curcumin inhibits HSC proliferation and activation and modulates the immune microenvironment. The protection of hepatocytes by reducing oxidative stress and inflammatory responses is also involved in the antifibrotic effects of some natural products, such as silibinin, silymarin, anthocyanins, and emodin and rhein. Finally, the strength of evidence for the antifibrotic effect of several natural products is different based on various liver fibrosis models. Most natural products, including flavonoids, quinones, lignans, and phenols and acids, present excellent antifibrotic function in CCl_4_, DMN, TAA, *Schistosome*, and ethanol-treated models. However, a few compounds, such as skullcapflavone I and magnolol, have only been confirmed to have effects on HSCs in vitro. Stronger evidence is needed for the effects of these compounds in liver fibrosis. Despite the rapid growth of studies on antifibrotic natural products, there are still future breakthroughs in two aspects. The potent effects of natural compounds have been presented, but the low bioavailability of several compounds vastly limits their clinical application. Enhancement of pharmacokinetic parameters for these compounds should be investigated. Moreover, the combined application of several natural products seems to be a promising method for liver fibrosis treatment. The combination of silymarin with caffeine, puerarin with vitamin D, baicalin with rosmarinic acid, and genistein with taurine and EGCG has been explored in previous studies. The results also show the potent synergistic effect of these combinations.

## 7. Conclusions

In summary, exploration on natural products against liver fibrosis is increasingly thorough. Natural products are a potential resource for the development of agents to treat liver fibrosis. Thus, natural products are very valuable when seeking novel therapeutic agents for liver fibrosis.

## Abbreviations

ECM: Extracellular matrix
HSCs: Hepatic stellate cells
HBV: Hepatitis B virus
NAFLD: Nonalcoholic fatty liver disease
NASH: Nonalcoholic steatohepatitis
ROS: Reactive oxygen species
LSECs: Liver sinusoidal endothelial cells
TGF-*β*: Transforming growth factor-*β*
TCM: Traditional Chinese medicine
HCC: Hepatocellular carcinoma
TAA: Thioacetamide
GSH: Glutathione
CTGF: Connective tissue growth factor
PPAR-*γ*: Peroxisomal proliferator-activated receptor *γ*
DMN: Dimethylnitrosamine
PDGF-BB: Platelet-derived growth factor-B subunit homodimer
LDH-A: Lactate dehydrogenase-A
ER: Endoplasmic reticulum
TIMP-1: Tissue inhibitor of metalloproteinases-1
LPS: Lipopolysaccharide
NDMA: N′-Nitrosodimethylamine
PKC: Protein kinase C
EGCG: Epigallocatechin-3-gallate
RAGE: Receptor for advanced glycation end-products
DLK1: Delta-like homolog 1
CBR1: Cannabinoid receptor type-1.
